# Study on the Difference in the Contribution of Soil Particle Sizes to Heavy Metal Exposure of Children Around Smelting Area

**DOI:** 10.3390/toxics14030253

**Published:** 2026-03-12

**Authors:** Ran Li, Jingzhi Yu, Xiaoli Duan, Beibei Wang, Dekang Liu, Liwen Zhang, Kai Yang, Hongguang Cheng

**Affiliations:** 1 School of Energy and Environmental Engineering, University of Science and Technology Beijing, Beijing 100083, China; m202320219@xs.ustb.edu.cn (R.L.);; 2 College of Water Sciences, Beijing Normal University, Beijing 100875, China

**Keywords:** soil, hand-loaded dust, particle size, heavy metals, bioaccessibility, health risk

## Abstract

This study focused on children residing near a smelter in Baiyin, and investigated the impact mechanism of different soil particle sizes on children’s exposure to heavy metals. By analyzing the distribution pattern of concentrations and bioaccessibilities of typical heavy metals (Cd, Cr, Cu, Ni, Pb) across four particle size fractions (<63 μm, 63–150 μm, 150–250 μm, 250–352 μm), and incorporating the size-selective adherence characteristics of children’s hand-loaded dust, this research quantitatively assessed the contribution of each particle size fraction to children’s health risks from oral exposure. The results showed that fine particle size soil (<63 μm) exhibited both higher concentration and bioaccessibility of heavy metals, which were 1.3–1.9 times and 1.1–2.2 times higher, respectively, than those of the coarsest fraction (250–352 μm). The proportion of particles < 63 μm in children’s hand-loaded dust (64.3%) was significantly higher than that in ambient soil, demonstrating selective adherence towards finer particles during children’s exposure. Due to the particle size-selective effects on metal concentration, bioaccessibility, and actual child exposure, fine soil particles constituted the primary source of heavy metal exposure risk via oral ingestion in children. Soil particles with a size of <63 μm contributed 48–60% to the total exposure risk of the five heavy metals. Therefore, in the health risk assessment of soil around smelting plants, the influence of particle size on the occurrence characteristics of metals, bioaccessibility, and children’s actual exposure behavior should be considered concurrently to enhance the accuracy and targetability of assessment and control measures.

## 1. Introduction

Metal smelting and processing are recognized as one of the top ten global pollution issues and serve as a key source of heavy metal contamination in the environment [1]. This process releases substantial amounts of heavy metals into the surrounding environment through pathways such as exhaust gas emissions, slag accumulation, and wastewater migration, leading to significantly elevated concentrations of pollutants such as Pb and Cr in soils [2]. These pollutants persist and accumulate in soil, posing long-term ecological risks [3]. More importantly, heavy metals in soil can be transferred to humans via the food chain or direct contact [4]. Among various exposure pathways, oral ingestion represents a significant route of human exposure to soil contaminants [5,6]. Children are especially susceptible to heavy metal exposure via oral ingestion of soil due to their behavioral patterns, such as frequent ground-level activities and hand-to-mouth contact, as well as their stage of physiological development, making their health risks particularly prominent. Meanwhile, extensive epidemiological and toxicological studies have confirmed the significant adverse health effects of heavy metal exposure, including poisoning, cancer, neurological damage, and impaired immune function [7,8,9]. Consequently, conducting health risk assessments focused on children’s oral ingestion of soil heavy metals is of critical importance.

Soil is a complex and heterogeneous system composed of particles of different sizes, in which particle size distribution serves as a key factor controlling the environmental fate of heavy metals [10,11]. Heavy metals are heterogeneously distributed among different soil components [12]. Studies have shown that most heavy metals tend to preferentially accumulate in fine particle fractions [13]. For example, Ma et al. investigated the distribution of Cd in soil particles of different sizes and found Cd contents in the order of clay (14.63 mg·kg^−1^) > fine silt (5.71 mg·kg^−1^) > coarse silt (2.75 mg·kg^−1^) > sand (1.27 mg·kg^−1^) [10]. Karnaet et al. found that the total Pb and As contents were higher in the smaller (<38 μm) soil particle fractions than in the larger (150–250 μm) fractions [14]. Furthermore, heavy metals typically exhibit higher bioaccessibility in fine particles [15,16,17]. For example, a study on contaminated suburban soils showed that the gastric-phase bioaccessibility of Pb increased as particle size decreased across the fractions < 50 μm, <100 μm, and <250 μm [18].

However, prevailing oral exposure risk assessment models typically use the total concentration of heavy metal in soil within a single particle-size range (e.g., the <150 μm recommended by the U.S. Environmental Protection Agency (U.S. EPA) [19,20] or the <250 μm suggested by the European BARGE method [21]) to represent the exposure level. This approach overlooks the size selectivity of the soil particles to which humans are actually exposed. Hand-to-mouth contact is the primary pathway for soil ingestion. Tsou et al. found that the hands are the primary site of skin contact with soil, compared to the forearms, feet, and lower legs [22]. The dust adhered to hands is generally considered representative of soil ingested via the oral route [23]. Studies have indicated that fine particles dominate the soil adhered to children’s hands. For example, Ikegami et al. found that particles < 63 µm accounted for 81.7% of the total mass of soil adhered to children’s hands [24]. Bergström et al. also noted that over 60% of the adhered particles had a diameter smaller than 63 μm following a desiccation test [25]. This suggests that relying on bulk soil concentrations within a single particle-size range for risk assessment may misrepresent the actual exposure contributions from different particle sizes. Specifically, such an approach could overestimate the risk attributable to coarse particles, which adhere less readily, while underestimating the risk from fine particles that are highly enriched with contaminants, more reactive, and more likely to adhere. Therefore, quantifying the coupled effects of size-specific exposure and size-specific contamination is crucial for enhancing the accuracy of health risk assessments for children. Yamamoto et al. demonstrated that finer soil particles preferentially adhere to children’s hands, making them the primary vehicle for oral ingestion [26]. Doyi et al. emphasized that incorporating particle size distribution and bioaccessibility into risk assessment substantially refines health risk estimates for trace elements in dust [16].

In summary, previous studies have predominantly examined heavy metal variations across different particle sizes, but have not systematically incorporated children’s behavioral patterns to address differential exposure to soil particles of different sizes. To address this research gap, this study focused on children living near a typical smelter as the research subject, with the following objectives: (1) To analyze the particle size distribution of children’s hand-loaded dust to clarify the selective adhesion characteristics of children towards soil particles of different sizes during hand-to-mouth contact; (2) To analyze the relationship between soil particle size and the distribution patterns of heavy metal concentrations and bioaccessibility, thereby clarifying the contamination characteristics specific to different particle sizes; (3) By integrating both datasets, to quantitatively assess the contribution rate of soil from each particle size fraction to the total exposure risk via oral ingestion for children, thereby identifying the key particle size ranges driving the risk.

## 2. Materials and Methods

### 2.1. Study Site and Subject Selection

**Figure 1:**
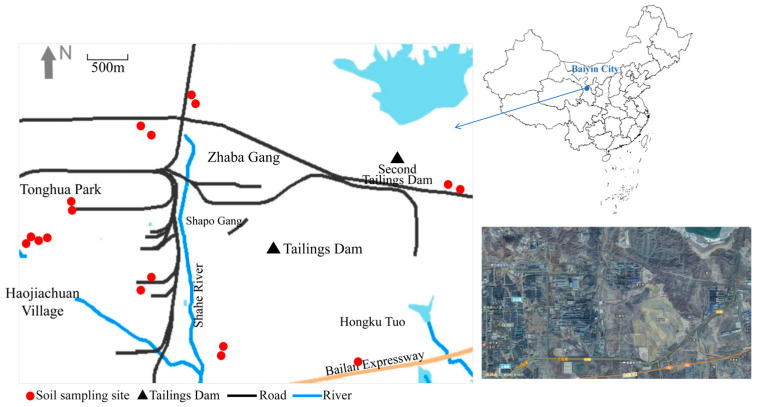
Map of the study area. The map shows the Baiyin smelter and surrounding residential areas in Gansu Province, China. The bottom-right inset shows a satellite image of the study area. Key geographic features are labeled in Chinese from left to right, top to bottom: Dongtai Primary School Baiyin District, Zhaba Gang, No. 2 Tailings Dam, Silong Road, Kunpengtai Building, Shahe River, Shapo Gang, Tailings Dam, Jinyu Park, Hongku Tuo, Baiyin Second People’s Hospital, Gongyuan Road, Lanbao Road, G109 National Highway, yaqushui Primary Schoo, G6 Expressway (Beijing–Lhasa Expressway), and Gaohuangya Cliff. Base image: Tianditu (www.tianditu.gov.cn), Map Number: GS (2024) 0568.

The study site is located in Baiyin City, Gansu Province (Figure 1). This area is situated in the central part of the Loess Plateau in northwestern China, with a long and extensive history of Cu-Zn-Pb ore mining. It serves as a crucial base for China’s non-ferrous metals industry. The area features a dry climate with scarce precipitation, high evaporation rates, and significant diurnal temperature variations [27]. The local soil belongs to the dry soil type of calcareous loam, with a high content of calcium carbonate, a slightly alkaline pH value, and a low organic matter content [28].

A total of 30 children aged 3–12 years residing within a 3 km radius of the smelting plant were randomly recruited as study participants, with a male-to-female ratio of 1:1. Soil samples were collected from the residential courtyards of these children, as these areas represent the primary sites of soil contact during children’s daily activities. In total 17 surface soil samples were collected, corresponding to the 30 participating children. In cases where multiple children resided in the same household or in adjacent dwellings sharing a common courtyard, a single composite soil sample was collected to represent that location. The spatial distribution of the 17 sampling sites is shown in Figure 1, covering the main residential areas surrounding the facility. The study was approved by the Ethics Review Committee of University of Science and Technology Beijing. Prior to sampling and investigation, written informed consent was obtained from all participants and their guardians.

### 2.2. Sample Collection and Pretreatment

Hand-loaded Dust: Hand-loaded dust samples were collected using an ultrapure water rinse method. This procedure was performed after outdoor activity and before handwashing. The specific procedure was as follows: 100 mL of ultrapure water was used to rinse the palms, backs of hands, and finger crevices, while the children were guided to rub repeatedly for 30–60 s, thereby ensuring that the dust particles were completely transferred to the rinsing liquid. The solution was then transferred through a funnel into clean, deionized-water-rinsed brown PVC bottles, sealed and stored at 4 °C until analysis.

Soil: Surface soil (0–5 cm) was collected from the courtyards where children reside, as these areas represent the primary sites of soil contact during children’s daily activities. A total of 17 composite soil samples were collected, corresponding to the 30 children, with multiple children sharing a common courtyard represented by a single sample. All sampling sites were located within a 3 km radius of the smelter, and GPS was used for the geographical positioning of the sampling points. At each sampling point, four subsamples were collected within a 5 m radius and combined to form a composite sample. Visible impurities such as plant roots and leaves were removed, and the samples were then passed through a 5 mm sieve, placed in polyethylene bags, and preserved.

The particle size distributions of hand-loaded dust and unsieved bulk soil were determined using a laser particle size analyzer (Mastersizer 3000, Malvern Panalytical Ltd., Malvern, Worcestershire, UK). To ensure consistency with the particle size of hand-loaded dust, the soil samples were first passed through a 352 µm nylon sieve. This matched the maximum particle size observed in the hand-loaded dust and excluded particles larger than 352 µm, which are unlikely to adhere to children’s hands. The sieved soil was then passed sequentially through 250 μm, 150 μm, and 63 μm nylon sieves. This process yielded four discrete size fractions: <63 μm, 63–150 μm, 150–250 μm, and 250–352 μm, which were collected separately for subsequent analysis of metal concentrations and bioaccessibility.

For hand-loaded dust, the particle size distribution was characterized using two complementary approaches. First, the mean mass fraction for each size fraction was determined. For each of the four size fractions (<63 μm, 63–150 μm, 150–250 μm, and 250–352 μm), the arithmetic mean of the mass fractions from the 30 children was calculated. These mean values were then normalized to sum to 100% to obtain the pooled particle size distribution used in the exposure assessment. Second, the overall median particle size (D50) for the study population was calculated as the arithmetic mean of the 30 individual D50 values obtained from the laser diffraction analysis.

Soil physicochemical properties were determined using standard methods. Soil pH was measured in a 1:2.5 (*w*/*v*) soil-to-water suspension using a pH meter (PHS-3C, Shanghai INESA Scientific Instrument Co., Ltd., Shanghai, China). Soil organic matter (SOM) content was determined by the loss-on-ignition method, where oven-dried soil samples were combusted at 550 °C for 4 h. The results of soil physicochemical analysis are summarized in Table S1.

### 2.3. Total Concentration of Heavy Metals

The determination of heavy metals in the soil followed the method described by Wei et al. [29]. Briefly, 0.15 g of each soil sample was weighed into a polytetrafluoroethylene (PTFE) digestion vessel. Then, 6 mL of nitric acid (HNO_3_, 68%, ρ ≈ 1.42 g/mL), 3 mL of hydrochloric acid (HCl, 37%, ρ ≈ 1.19 g/mL), and 2 mL of hydrofluoric acid (HF, 40%, ρ ≈ 1.16 g/mL) were added sequentially. Detailed information on the reagents used for microwave digestion, including chemical formulas, purity, specifications, and manufacturers, is provided in Table S2. Ultrapure water (resistivity > 18 MΩ·cm) was prepared in our laboratory. The sealed vessels were subjected to microwave digestion (MARS-5, CEM Corporation, Matthews, NC, USA) according to the temperature programme shown in Table S3. The concentrations of Cd, Cr, Cu, Ni, and Pb in the filtrate were determined using inductively coupled plasma mass spectrometry (ICP-MS 8900, Agilent Technologies, Santa Clara, CA, USA). Each soil sample was digested in triplicate (*n* = 3 analytical replicates). Method blanks and certified reference materials (CRM, GBW07402a, Institute of Geophysical and Geochemical Exploration, Langfang, China) were included in each digestion batch to ensure quality control. Multi-element calibration standards were prepared by diluting a stock standard solution (1000 mg/L, National Institute of Metrology, Beijing, China) with 5% HNO_3_. A six-point calibration curve was established for each metal at concentrations of 0, 1.0, 5.0, 10.0, 20.0, and 50.0 μg/L. The linearity of the calibration curve was evaluated by the correlation coefficient (R^2^), which was consistently >0.999 for all target metals. A calibration standard was re-analyzed after every 20 samples to monitor instrumental drift, and the relative percent difference was maintained below 10%. Internal standards (^72^Ge, ^115^In, and ^209^Bi) were used to correct for matrix effects and instrumental instability. The limits of detection (LOD) for Cd, Cr, Cu, Ni, and Pb were 0.03, 2.00, 0.70, 2.00, and 1.00 mg·kg^−1^, respectively.

### 2.4. Bioaccessibility of Heavy Metals

The bioaccessibility of heavy metals was determined using the physiologically based extraction test method (PBET) following established protocols [30]. This method simulates the absorption process of heavy metals in the human gastric and intestinal digestive systems through in vitro simulation of digestive fluids [31,32]. It has been widely validated for assessing metal bioaccessibility in contaminated soils [30,33]. Subsequent studies have demonstrated strong correlations between PBET results and in vivo animal models for multiple metals, including As, Cd, and Pb [31,34,35,36], supporting its applicability for human health risk assessment.

Simulated gastric fluid was prepared by dissolving 1.25 g of pepsin, 0.5 g of sodium malate, 0.5 g of sodium citrate, and 420 μL of lactic acid in an appropriate volume of deionized water, adjusting the volume to 1 L, and then regulating the pH to 2.5 ± 0.1 with hydrochloric acid. Precisely 0.5 ± 0.0001 g of soil sample was weighed, mixed with 50 mL of the simulated gastric fluid, and digested in a constant-temperature water bath shaker (SHA-C, Changzhou Guohua Electric Appliance Co., Ltd., Changzhou, China) at 37 °C and 150 rpm for 1 h. The pH was adjusted every 15 min to maintain it at 2.5 ± 0.1. After digestion, 5 mL of the supernatant was collected as the gastric-phase digest for analysis. Subsequently, the pH of the remaining mixture was adjusted to 7.0 ± 0.1, followed by the addition of 0.025 g of trypsin, 0.0875 g of bile salts, and 0.025 g of pancreatin. The mixture was shaken thoroughly for 4 h while maintaining the pH. Finally, 5 mL of supernatant was collected as the intestinal-phase digestate for measurement. The reagents required for the PBET in this study, along with their specifications and brands, are shown in Table S4. The ultrapure water (resistivity > 18 MΩ·cm) used was prepared in-house. The PBET assay was performed in triplicate for each soil sample (*n* = 3 experimental replicates). Blank samples (without soil) were included in each batch to monitor background contamination. Both gastric and intestinal phase digestion samples were centrifuged (TG16-WS, Xiangyi Centrifuge Instrument Co., Ltd., Changsha, China) and filtered through a 0.45 μm membrane filter (Jinteng Experimental Equipment Co., Ltd., Tianjin, China). The concentrations of Cd, Cr, Cu, Ni, and Pb in the filtrate were measured using ICP-MS. The bioaccessibility (*BA*) of heavy metals in the soil via oral ingestion in the human gastric and intestinal phases was calculated by Equation (1):

(1) $$BA=\left(\frac{CV}{TM}\right)\times 100\%$$

where *C* is the soluble concentration of heavy metals in the gastric or intestinal phase reaction solution (mg·L^−1^); *V* is the volume of reaction solution in each reactor (L); *T* is the total content of heavy metal in the soil (mg·kg^−1^); *M* is the mass of soil sample added to the reactor.

### 2.5. Health Risk Assessment Model for Oral Ingestion

#### 2.5.1. Exposure Assessment

Based on the exposure assessment model recommended by the U.S. EPA [9,37], the average daily dose (*ADD_i_*) of a target metal ingested orally from soil of different particle sizes was calculated using Equation (2):

(2) $${ADD}_{i}=\frac{C_{i}\times {IR}_{S}\times CF\times {BA}_{i}\times EF\times ED}{BW\times AT}$$

where *C_i_* is the concentration of the target metal in soil in the i-th particle size range (mg·kg^−1^); *BA_i_* is the bioaccessibility of the target metal in soil in the i-th particle size range, expressed as a unitless fraction. *IR_S_* is the soil ingestion rate. *CF* is the mass conversion factor (1 × 10^−6^ kg·mg^−1^). *EF* is the exposure frequency. *ED* is the duration of exposure. *BW* is body weight. *AT* is the average exposure time.

The total exposure dose (*ADD*) of the target metal across all particle sizes was then computed via Equation (3).

(3) $$ADD=\sum_{i=1}^{n}{\Delta m_{i}\times ADD}_{i}$$

where Δ*m_i_* is the mass fraction of hand-loaded dust in the i-th particle size range, expressed as a unitless fraction.

It is important to clarify that, in the exposure equations, the metal concentrations (*Cᵢ*) and bioaccessibility (*BAᵢ*) used in the *ADDᵢ* calculation for each size fraction are derived from soil samples, representing the environmental contaminant source. In contrast, the exposure weighting used the particle size distribution of hand-loaded dust, based on the established finding that finer particles preferentially adhere to children’s hands and thus represent the actual particles ingested via hand-to-mouth activity [26,38].

#### 2.5.2. Health Risk Assessment

This study focused on the non-carcinogenic risks via oral ingestion of heavy metals. The hazard quotient (*HQ*) for the target metals in each particle-size range was calculated using Equation (4).

(4) $$HQ=\frac{ADD}{RfD}$$

where *RfD* is the oral ingestion reference dose (mg·(kg·d)^−1^). The *RfD* of Cd, Cr, Cu, Ni and Pb are 1 × 10^−3^, 3 × 10^−3^, 4 × 10^−3^, 2 × 10^−3^, and 1.4 × 10^−3^ mg·(kg·d)^−1^, respectively [9,39,40,41]. For Pb, a hazard quotient approach was used to maintain consistency with the risk characterization framework applied to other metals (Cd, Cr, Cu, Ni) and to allow direct comparison of relative risks across contaminants. The oral *RfD* for Pb was obtained from the U.S. EPA’s Risk Assessment Guidance for Superfund (RAGS), Volume I-Human Health Evaluation Manual (Part A) [42]. For Cr, the toxicity value used in this study corresponds to Cr(VI) obtained from the U.S. EPA Integrated Risk Information System (IRIS). However, as our measurements represent total Cr, the resulting risk estimates for Cr are likely conservative.

Based on the above equations and parameters, a deterministic risk assessment was first conducted to obtain point estimates of health risks. In this approach, the mean concentrations and mean bioaccessibility of heavy metals for each particle size fraction, together with a fixed ingestion rate of 103 mg·d^−1^, were used as input values to calculate the *HQ* for each metal and particle size fraction. This fixed ingestion rate represents the most likely value of a triangular distribution (minimum: 66 mg·d^−1^; maximum: 161 mg·d^−1^) derived from *Exposure Factors Handbook of Chinese Population*.

The probabilistic health risk assessment for the non-carcinogenic risks of Cd, Cr, Cu, Ni, and Pb was conducted using Monte Carlo simulation with 10,000 iterations. The *RfD* and temporal exposure parameters (exposure frequency, exposure duration, and averaging time) were typically treated as deterministic values [43,44]. Data on ingestion rates and average body weight were obtained from *Exposure Factors Handbook of Chinese Population* [45]. In the Monte Carlo simulation, the hand-loaded dust mass fractions (Δ*mᵢ*) were treated as fixed values (i.e., the normalized arithmetic means derived from the 30 children), as the variability in these mass fractions was relatively low. In contrast, metal concentrations and bioaccessibility exhibited substantially higher variability and thus represented the primary sources of uncertainty in the risk assessment. Accordingly, probability distributions were fitted to the metal concentrations and bioaccessibility values for each of the four size fractions using data from the soil samples. Goodness-of-fit was assessed using the Anderson-Darling test (Table S5). Correlations among input parameters were not explicitly considered in the primary analysis due to the limited sample size (*n* = 17) for reliable correlation estimation. Table S6 summarizes the probability distribution models of the exposure parameters used in the probabilistic health risk assessment.

### 2.6. Quality Control

To ensure data quality, a systematic quality control procedure was implemented in the experiment. During the sample pretreatment stage, guaranteed grade reagents were used, all containers were acid-washed, and duplicate samples (accounting for 10% of each batch) were analyzed to monitor procedural precision. In the instrumental analysis stage, background contamination was monitored by setting reagent blanks and laboratory blanks. The national certified reference material (GBW07402a) was used to verify the accuracy of the method. The recoveries of Cd, Cr, Cu, Ni, and Pb were 93.6%, 97.5%, 102%, 106%, and 99.5%, respectively. All recovery rates were within the acceptable range of 85–115%. For ICP-MS analysis, the standard curve was checked after every 20 samples were detected, and the relative deviations were maintained below 10% for recalibration checks and 30% for blanks, respectively. In the data processing stage, the corresponding blank value was deducted from all test results. The data below method’s limit of detection were replaced by 1/√2 of the detection limit. All results are reported as the mean ± standard deviation of triplicate measurements. The relative standard deviation (*RSD*) for replicate analyses was consistently below 10%, and the coefficients of variation in parallel samples were all below 5%, indicating good reproducibility and that the analytical precision met the quality control requirements.

### 2.7. Statistical Analysis

IBM SPSS Statistics (version 25.0, IBM Corp., Armonk, NY, USA) was used to analyze the data differences between soil particle size and soil heavy metal content and bioaccessibility. The Monte Carlo simulations were performed using Crystal Ball software (version 11.1.2.4, Oracle Corporation, Redwood Shores, CA, USA) integrated in Microsoft Excel (Microsoft Corporation, Redmond, WA, USA). OriginPro software (2021 version, OriginLab Corporation, Northampton, MA, USA) was used to draw the charts in this study.

## 3. Results and Discussion

### 3.1. Particle Size Distribution of Hand-Loaded Dust

**Figure 2:**
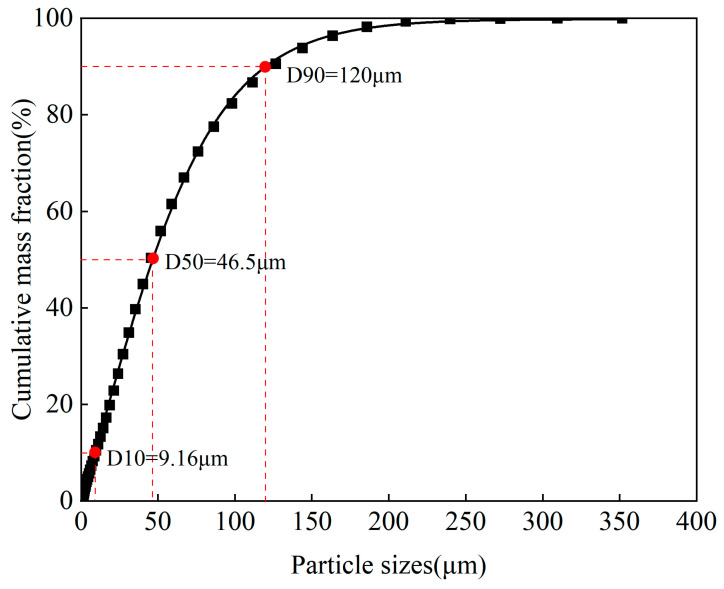
Cumulative mass distribution of particle sizes in hand-loaded dust (D10, D50, and D90 correspond to the cumulative frequencies of 10%, 50%, and 90%). The black squares represent individual experimental data points.

The cumulative mass distribution of particle sizes in children’s hand-loaded dust is shown in Figure 2. The particle size distribution of children’s hand-loaded dust ranged from 0 to 352 μm, with a median particle size of 46.5 μm. Particles smaller than 63 μm constituted the largest fraction, accounting for 64.3% of the total mass. Particles with diameters of 63–150 μm, 150–250 μm, and 250–352 μm accounted for 30.9%, 4.31%, and 0.490%, respectively, with the last group showing the smallest proportion. The median particle size of hand-loaded dust measured in this study fell within the range (33–150 μm) reported in previous studies [24,26,38,46,47].

Compared to the soil from children’s courtyards, the particle size composition differed significantly (Figure S1). The soil particle size of the activity site was wider (0–756 μm), with particles <63 μm, 63–150 μm, and 150–250 μm accounting for 44.6%, 30.9%, and 14.9% of the total mass, respectively. The significant difference in particle size composition between hand-loaded dust and soil suggests that the adherence of children’s hands to soil particles is significantly particle size selective, resulting in a high enrichment of fine particles (<63 μm) in hand-loaded dust (Figure S2) [23,48]. This selective enrichment is related to the larger specific surface area and surface energy of fine-grained soil, which makes it more likely to be captured and retained on the skin due to stronger intermolecular forces such as van der Waals forces generated when it contacts the skin [26,38]. This filtering effect indicates that assessing child exposure directly using a single particle size range of ambient soil may underestimate the actual exposure to fine soil particles.

### 3.2. Variation in Metal Concentrations with Soil Particle Size

The average concentrations of Cd, Cr, Cu, Ni and Pb in the soil were 18.5, 67.9, 565, 26.2, and 432 mg·kg^−1^, respectively. The concentration differences among elements were significant, with Cu and Pb showing the highest levels, exceeding the other elements by an order of magnitude. Compared to the background levels of soil in Gansu Province, the average concentrations of all elements except Cr and Ni were higher than their respective background levels by factors ranging from 23.0 to 160 [49,50]. However, the contamination levels of all elements remained below both the risk screening values and intervention thresholds for Class II land in the Standard for Soil Pollution Risk Management and Control of Construction Land (GB 36600-2018) (pH > 7.5). Compared with studies on soils surrounding smelters, the levels of all elements in this study were within previously reported ranges: Cd (0.06–99.1 mg·kg^−1^), Cr (11.4–287 mg·kg^−1^) [27], Cu (33.7–587 mg·kg^−1^), Ni (4.30–49.4 mg·kg^−1^), and Pb (30.5–2179 mg·kg^−1^) [51].

**Figure 3:**
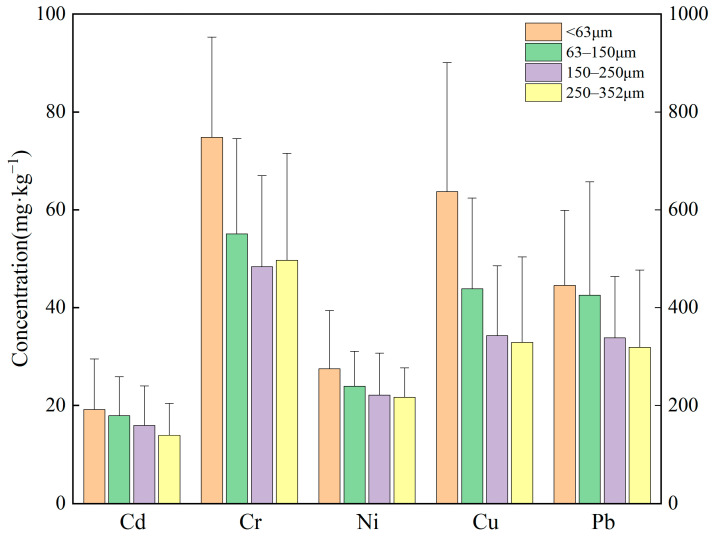
Concentration of heavy metals across different particle sizes (Cd, Cr, and Ni correspond to the left vertical axis. Cu and Pb correspond to the right vertical axis). The vertical error bars represent the standard deviation (SD) of replicate measurements.

The concentration distribution characteristics of heavy metals in soil with different particle sizes were shown in Figure 3. In general, the concentrations of Cd, Cr, Cu, Ni and Pb increased as particle size decreased, with the heavy metal content of the <63 μm fraction being 1.3–1.9 times higher than that of the 250–352 μm fraction. Statistical analysis revealed that significant differences in concentration among the size fractions were observed only for Cr (Kruskal–Wallis, *p* = 0.003). Pairwise comparisons with Bonferroni correction showed that the concentration of Cr in the <63 μm fraction was significantly higher than those in all the other fractions (*p* < 0.01 for all comparisons). For Cd, Cu, Ni, and Pb, although a trend of increasing concentrations with decreasing particle size was observed, no significant differences were found among the size fractions (*p* > 0.05). This enrichment in fine particles is consistent with their larger specific surface area and higher content of reactive components such as organic matter and Fe/Mn oxides, which facilitate metal adsorption and retention [52,53,54,55]. In addition, heavy metals emitted from smelters predominantly enter the environment as aerosols or fine particles and subsequently settle, readily mixing with intrinsic fine soil constituents and further exacerbating their accumulation in the fine particle fraction [56,57].

For specific elements, the concentration-particle size distribution patterns differed among heavy metals. Concentrations of Cd and Pb decreased continuously with increasing particle size. The concentrations of Cr and Cu decreased slowly within fine particle size range (<250 μm), but decreased significantly in the coarse particle size range (250–352 μm). The concentration of Ni decreased with the increase in particle size within the range of <250 μm, and then tended to stabilize. This trend was mainly attributable to the difference in sources of the heavy metals. Based on the results of source analysis of the existing literature in this research area, Cd and Pb were found to originate primarily from smelting emissions [56,58,59]. These anthropogenic heavy metals largely deposit in the environment primarily as fine particles, where they readily form complexes with organic matter in fine soil particles and become immobilized, resulting in significant enrichment in the fine fraction [60]. In contrast, although Cu was also of anthropogenic origin, its distribution pattern differed. A portion of the Cu contamination in the study area derived from waste residues generated during Cu mining and mineral processing [28]. These waste-slag particles were relatively coarse and entered the soil through mechanical mixing and localized deposition, thereby weakening the absolute enrichment of Cu in the fine fraction and leading to a more gradual decrease in concentration within the <250 μm size range. Cr and Ni are mainly from natural sources, occurring within stable mineral lattices, resulting in more uniform distribution across particle sizes [61].

### 3.3. Variation in Metal Bioaccessibilities with Soil Particle Size

**Figure 4:**
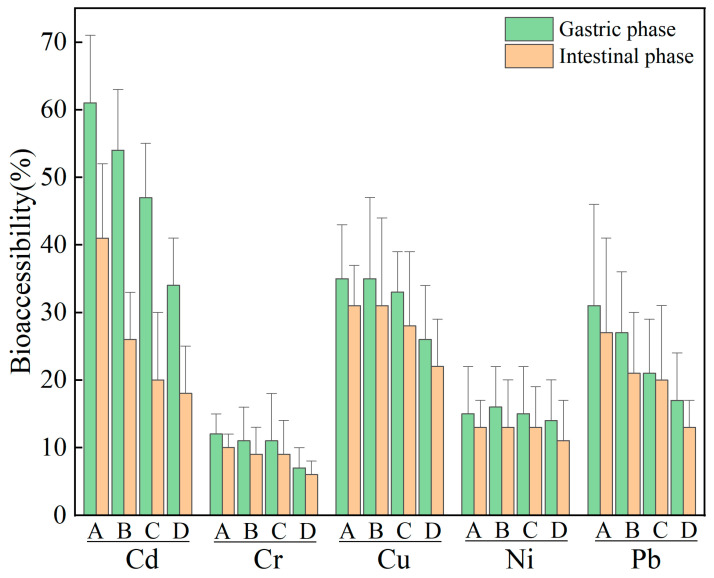
Bioaccessibility of heavy metals in the gastric phase and intestinal phase across different particle sizes (A: <63 μm; B: 63–150 μm; C: 150–250 μm; D: 250–352 μm). The vertical error bars represent the standard deviation (SD) of replicate measurements.

The relationship between bioaccessibility and particle size for heavy metals in the gastrointestinal phase was shown in Figure 4. The average bioaccessibilities of Cd, Cr, Cu, Ni, and Pb in the gastric phase were 49.1%, 9.93%, 32.1%, 14.9%, and 24.1%, respectively. During the intestinal phase, these values decreased to 26.3%, 8.61%, 28.0%, 12.6%, and 20.1%, respectively. In general, Cd and Cu exhibited significantly higher bioaccessibility compared to the other metals, with Cr showing the lowest values. Furthermore, the bioaccessibility of all elements was consistently higher in the gastric phase than in the intestinal phase. The difference was primarily related to the sources, chemical forms, and dissolution behaviors of heavy metals in the soil and gastrointestinal fluids [62]. Studies have shown that elements of anthropogenic origin such as Cd, Cu, and Pb, which were often associated with more labile forms, were more readily released in the acidic gastric environment. In contrast, elements that are mainly derived from natural sources and dominated by stable forms, such as Cr, exhibit low desorption potential [10]. The transition in pH of the simulated digestive fluid from strongly acidic in the gastric phase to neutral or weakly alkaline in the intestinal phase was a key environmental factor contributing to the observed decrease in bioaccessibility. The acidic gastric environment favors the desorption and dissolution of heavy metals from soil particles. Upon entering the intestinal phase, the rise in pH promotes processes such as precipitation, adsorption, or speciation transformation for certain metals, reducing their solubility and consequently lowering their bioaccessibility. Furthermore, ions such as Cd tend to form hydroxide precipitates or become strongly adsorbed onto solid surfaces as pH increases, whereas Cu can form soluble complexes with components like bile salts in intestinal fluid, allowing it to maintain a relatively high release rate even in the intestinal phase [63].

The bioaccessibility of the five heavy metals decreased gradually as the particle size increased. The lowest bioaccessibility values of all elements were found in the coarsest particle size category (250–352 μm). Bioaccessibility varied significantly with particle size for Cd (*p* < 0.001), Cr (*p* = 0.031), and Pb (*p* = 0.004) based on the Kruskal–Wallis test. Post hoc comparisons with Bonferroni correction showed that for Cd, bioaccessibility in the <63 μm fraction was significantly higher than that in all other fractions (*p* < 0.001). For Cr, bioaccessibility in the 63–150 μm fraction was significantly higher than that in the 250–352 μm fraction (*p* = 0.007). For Pb, bioaccessibility in both the <63 μm and 63–150 μm fractions was significantly higher than that in the 250–352 μm fraction (*p* = 0.002 and *p* = 0.005, respectively). No significant differences were observed for Cu (*p* = 0.056) or Ni (*p* = 0.958). Overall, the bioaccessibility of the five elements was approximately 40 to 60% lower in the coarsest particle size than in the finest particle size. This trend of bioaccessibility with particle size was closely related to the total concentration and chemical form distribution of heavy metals in the soil [15,64,65]. In this study, the total concentrations of all five metals decreased with increasing particle size, and bioaccessibility was generally positively correlated with total metal content, which partly explains the synchronous variation in these two parameters with particle size. Furthermore, previous studies have shown that as soil particle size decreases from 150–250 μm to <63 μm, the contents of more bioaccessible forms (such as carbonate-bound and exchangeable fractions) increase [62,66], further enhancing the release potential of heavy metals from fine particles.

The variation in bioaccessibility with particle size exhibited distinct patterns among elements. Specifically, the bioaccessibility of Cd and Pb consistently decreased with increasing particle size across all fractions. For Cu, however, it remained relatively stable in the fine fraction (<150 μm) but gradually declined with increasing particle size in the >150 μm range. For Ni and Cr, bioaccessibility decreased slowly within the <250 μm fraction and reached the lowest values in the coarse fraction (250–352 μm). This variation was largely influenced by differences in both the total heavy metal content and their chemical speciation. As the particle size decreases from 250 μm to 150 μm and then to <63 μm, the proportion of Cd and Pb in the exchangeable state increase accordingly. In fine particles < 63 μm, the proportion of Pb in exchangeable and oxide-bound states was significantly higher than that in coarser particles [66]. Therefore, fine particles not only have a higher total concentration but also a larger proportion of bioavailable forms, resulting in a significant increase in their bioaccessibility as the particle size decreases. The relatively stable bioaccessibility of Cu in the fine fraction was attributed to its consistent speciation distribution across these particle sizes [66]. In contrast, Ni and Cr are predominantly present in the stable residual fraction across all particle sizes [67]. In particular, Cr readily forms insoluble chromite-like minerals with iron and aluminum, resulting in extremely low solubility in simulated gastrointestinal fluids and consequently low bio accessibility which is less influenced by particle size.

### 3.4. Variation in Metal Exposure Risk with Soil Particle Size

A preliminary estimate of the non-carcinogenic risks associated with oral ingestion of heavy metals via different soil particle sizes, based on mean values, was presented in Table 1. This estimate considered both the intestinal bioaccessibility of heavy metals in soil and the mass distribution of hand-loaded dust to which children are actually exposed. The *HQ* is used to quantify the non-carcinogenic risk of a single metal. An *HQ* < 1 indicates that the estimated exposure dose is below the toxicological reference dose, suggesting a relatively low risk of adverse health effects, whereas an *HQ* > 1 implies that the exposure dose exceeds the reference dose, indicating a potential toxicity risk. In this study, the total non-carcinogenic risks were below 1, falling within the acceptable range. Among the elements, Pb posed the highest non-carcinogenic risk, followed by Cu and Cd, accounting for 59.2%, 32.8%, and 4.90% of the total Hazard Index, respectively (Figure S3). This is attributed to Pb exhibiting both high concentration and high bioaccessibility, coupled with a relatively low reference dose and significant non-carcinogenic effects, which collectively resulted in its highest non-carcinogenic risk among the five heavy metals.

**Figure 5:**
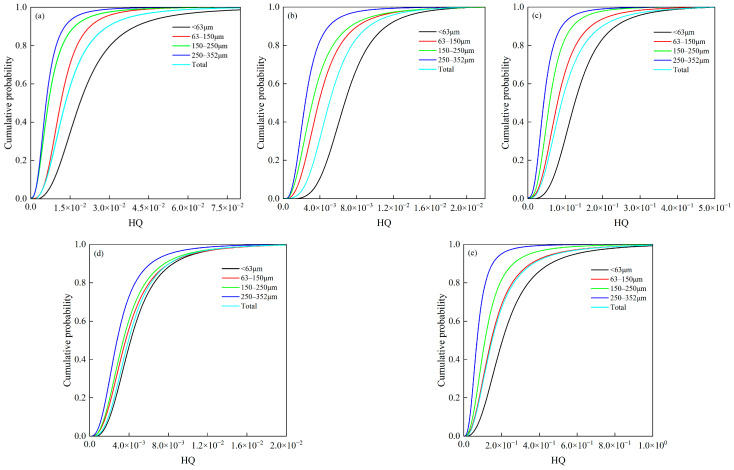
Cumulative probability distribution of non-carcinogenic risks across different particle sizes of heavy metals. Subplots (**a**–**e**) correspond to Cd, Cr, Cu, Ni, and Pb, respectively.

Figure 5 illustrates the hazard quotients of the five heavy metals across different particle sizes. The *HQ* for the five heavy metals in children’s oral ingestion increased with decreasing of particle size, indicating that fine particles are the primary contributors to exposure risk. Quantitative analysis showed that the non-carcinogenic risk levels of Cd, Cr, Cu, Ni, and Pb in the <63 μm particle size were 1.5–3.1 times those in particle size of 250–352 μm fraction, respectively. Regarding the contribution of different size fractions, the oral exposure health risks calculated based on the full particle size range of hand-loaded dust (0–352 μm) were higher than those based solely on the 63–150 μm fraction, but lower than those based solely on the <63 μm fraction. These findings suggest that using only the 63–150 μm fraction as the representative particle size for children’s exposure assessment may underestimate the actual risk, while focusing exclusively on the <63 μm fine particles may overestimate the overall exposure level. Therefore, weighting calculations based on the actual particle size distribution of hand-loaded dust provide a more accurate representation of the integrated risk from children’s oral exposure.

**Figure 6:**
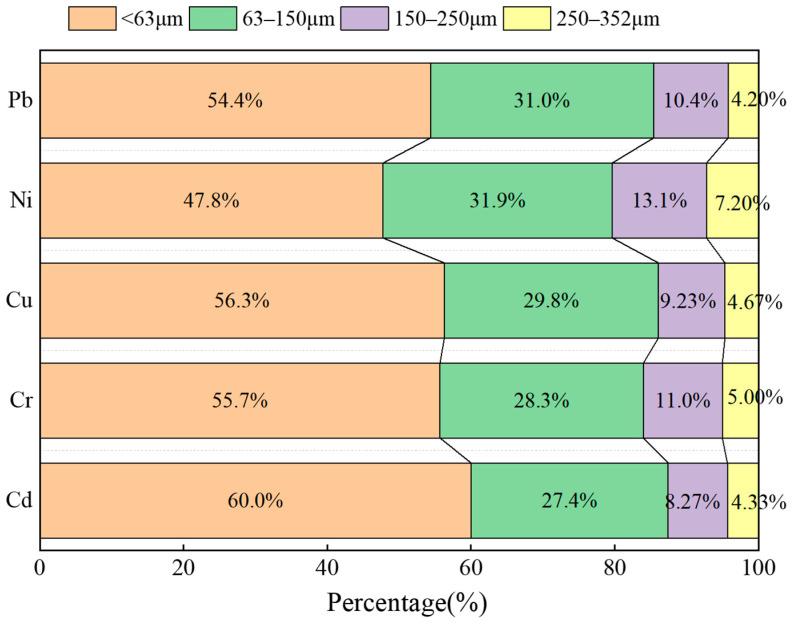
Risk contribution rate of heavy metals in soil with different particle sizes.

The contributions of different particle-size ranges to the non-carcinogenic risk from oral ingestion of heavy metals by children were illustrated in Figure 6. Fine particles (<63 μm) dominated the health risk contributions for all five heavy metals, followed by the 63–150 μm fraction, with their combined contribution exceeding 75% (Pb 85.4%, Ni 79.7%, Cu 86.1%, Cr 84.0%, Cd 87.4%). This indicates that focusing on particles < 150 μm for assessing children’s oral exposure risk is scientifically valid and consistent with existing research findings [10]. The overall risk contribution decreased progressively with increasing particle size, further confirming the pattern that “the finer the soil particles, the higher the associated health risk”. This distribution pattern resulted from the combined effects of heavy metal concentration, bioaccessibility, and the amount of dust adhered to hands. Notably, Cd showed a particularly prominent health-risk contribution from fine particles (<63 μm), primarily for three reasons: first, the concentration of Cd in the <63 μm fraction was significantly higher than that of other metals; second, its bioaccessibility in this size fraction was also markedly higher (approximately 1.2–1.3 times) than in coarser fractions; and third, the propensity of fine particles to adhere to hands further increased the likelihood of oral ingestion by children. In contrast, the concentration and bioaccessibility of Ni were the most uniformly distributed across particle sizes, resulting in the most gradual change in its risk contribution with increasing particle size.

This study has several limitations that should be considered when interpreting the results. First, the use of a *HQ* approach for Pb represents a methodological limitation. Regulatory guidance recommends blood Pb modeling (the IEUBK model) rather than the traditional *HQ* approach for Pb risk assessment. The *HQ* method based on the *RfD* assumes a threshold below which no effects are expected, which may not fully capture the toxicokinetic behavior of Pb. Consequently, the Pb risk estimates presented in this study should be interpreted as screening-level indicators rather than definitive predictions of health risk. Future studies should consider applying site-specific blood Pb modelling to refine Pb risk characterization in this population. Second, a significant source of uncertainty arises from the lack of Cr speciation data. In this study, total Cr concentrations were combined with a toxicity value for Cr(VI), which likely overestimates the actual oral risk, as a substantial fraction of the measured total Cr may be present as the less toxic Cr(III). To reduce this uncertainty, future investigations should include speciation analysis to quantify the proportion of Cr(VI) in site soils. Until such data become available, the Cr risk estimates reported here should be regarded as upper-bound screening estimates.

## 4. Conclusions

This study systematically investigated the characteristics of hand-loaded dust among children residing near a smelter in Baiyin, as well as variations in heavy metal concentrations and bioaccessibilities across different soil particle sizes. It further quantified the influence of soil particle size on children’s oral exposure risk to heavy metals. The results demonstrate that the particle size range of soil orally ingested by children was 0–352 μm. Fine particles (<63 μm) exhibited the highest heavy metal concentrations, with levels 1.3–1.9 times higher than those in the coarse fraction (250–352 μm). Bioaccessibility in the finest fraction was also substantially elevated, being 40–60% higher than in the coarsest fraction. These fine particles contributed the most to health risk, with Cd showing the highest contribution (60.0%) in the <63 μm fraction. Consequently, these fine particles represent the primary source of oral heavy metal exposure in children. Among the five metals, Pb and Cu exhibited relatively prominent exposure risks due to their enrichment in fine particles. These findings highlight the important role of fine soil particles in children’s exposure to heavy metals and suggest that future health risk assessments of contaminated sites should consider particle size-specific analysis. Particular emphasis should be placed on strengthening environmental management and exposure interventions targeting fine-particle soil, thereby providing a scientific basis for the precise prevention and control of health risks in children.

## Figures and Tables

**Table 1:** Mean-based hazard quotients for heavy metals by soil particle size fraction.

Particle Sizes	Cd	Cr	Cu	Ni	Pb
<63 μm	0.0210	0.00666	0.132	0.00478	0.229
63–150 μm	0.0125	0.00441	0.0908	0.00415	0.170
150–250 μm	0.00851	0.00387	0.0640	0.00387	0.129
250–352 μm	0.00667	0.00265	0.0483	0.00319	0.0790
Total	0.0149	0.00523	0.100	0.00430	0.183

## Data Availability

The original contributions presented in this study are included in the article/Supplementary Material. Further inquiries can be directed to the corresponding author.

## Supplementary Materials

The following supporting information can be downloaded at: https://www.mdpi.com/article/10.3390/toxics14030253/s1. Figure S1. Cumulative mass distribution of particle sizes in soil; Figure S2. Stacked bar chart of particle size contribution rates for hand-loaded dust and bulk soil; Figure S3. Contribution of soil heavy metals to total hazard quotient across different particle sizes; Table S1. Soil physicochemical properties; Table S2. Reagents required for soil metal analysis; Table S3. Microwave digestion temperature ramp program; Table S4. Reagents required for PBET; Table S5. Fitted distribution parameters and goodness-of-fit test results for soil concentrations and bioaccessibilities; Table S6. Probability distribution of health risk assessment parameters.
