# Supplementary material for: Study on the Difference in the Contribution of Soil Particle Sizes to Heavy Metal Exposure of Children Around Smelting Area
Source: Toxics. 2026 Mar 12;14(3):253. doi: 10.3390/toxics14030253 (PMC13030126; doi:10.3390/toxics14030253)
Supplement: Supplementary file 1 [file toxics-14-00253-s001.zip › toxics-4153314-supplementary.pdf]

Study on the difference in the contribution of soil particle sizes to heavy metal exposure of children around smelting area

Ran Li [1], Jingzhi Yu [2], Xiaoli Duan [1], Beibei Wang [1]*, Dekang Liu [1], Liwen Zhang [1], Kai Yang [2], Hongguang Cheng [2]

1.School of Energy and Environmental Engineering, University of Science and Technology Beijing, Beijing 100083, China;

2. College of Water Sciences, Beijing Normal University, Beijing 1000875, China

* Corresponding author: Beibei Wang.

E-mail address: wangbeibei723@163.com.

This supporting information provides texts and figures addressing.

Table S1. Soil physicochemical properties.

Table S2. Reagents required for soil metal analysis.

Table S3. Microwave digestion temperature ramp program.

Table S4. Reagents required for PBET.

Table S5. Fitted distribution parameters and goodness-of-fit test results for soil concentrations and bioaccessibilities.

Table S6. Probability distribution of health risk assessment parameters.

Figure S1. Cumulative mass distribution of particle sizes in soil.

Figure S2. Stacked bar chart of particle size contribution rates for hand-loaded dust and bulk soil.

Figure S3. Contribution of soil heavy metals to total hazard quotient across different particle sizes.

Table S1. Soil physicochemical properties.

	pH	*SOM*(g/kg)	*Total Al*(g/kg)	*Total Fe*(g/kg)	*Total Mn*(g/kg)
Mean	7.77	10.57	57.7	27.3	0.58
Standard Deviation	0.73	6.58	5.28	6.28	0.11

Table S2. Reagents required for soil metal analysis.

Reagent	Chemical Formula	Purity	Specification	Manufacturer
Nitric acid	HNO_3	Guaranteed reagent (GR)	500 mL	Aladdin
Hydrochloric acid	HCl	Guaranteed reagent (GR)	500 mL	Aladdin
Hydrofluoric acid	HF	Guaranteed reagent (GR)	500 mL	Aladdin

Table S3. Microwave digestion temperature ramp program.

Ramp time(min)	*Digestion temperature*(℃)	*Hold time*(min)
10	25→120	10
8	120→160	15
8	160→200	25

Table S4. Reagents required for PBET.

Reagent	Chemical Formula	Purity	Specification	Manufacturer
Pepsin	-	Guaranteed reagent (GR)	100 g	Aladdin
Sodium malate	$C_4H_4O_5Na_2 \cdot H_2O$	Guaranteed reagent (GR)	500 g	Aladdin
Sodium citrate	$C_6H_5Na_3O_7$	Guaranteed reagent (GR)	100 g	Aladdin
lactic acid	$C_3H_6O_3$	Guaranteed reagent (GR)	500 mL	Aladdin
Trypsin	$C_6H_{15}O_{12}P_3$	Guaranteed reagent (GR)	50 g	Aladdin
Pancreatin	-	Guaranteed reagent (GR)	50 g	Aladdin
Bile salts	-	Guaranteed reagent (GR)	100 g	Aladdin

Table S5. Fitted distribution parameters and goodness-of-fit test results for soil concentrations and bioaccessibilities.

Metal		Particle size	distribution	mean	Std	A-D	P value
Cd	*C*	<63 μm	Normal distribution	19.15	10.36	0.669	0.0610
		63–150 μm	Lognormal distribution	17.94	7.92	0.322	0.499
		150–250 μm	Lognormal distribution	15.94	8.09	0.606	0.613
		250–352 μm	Lognormal distribution	13.88	6.54	0.502	0.140
	BA	<63 μm	Lognormal distribution	0.41	0.11	0.370	0.193
		63–150 μm	Lognormal distribution	0.26	0.07	0.389	0.142
		150–250 μm	Normal distribution	0.2	0.1	0.411	0.302
		250–352 μm	Lognormal distribution	0.18	0.07	0.272	0.366
Cr	*C*	<63 μm	Normal distribution	74.83	20.48	0.641	0.092
		63–150 μm	Normal distribution	55.06	19.47	0.353	0.417
		150–250 μm	Normal distribution	48.38	18.58	0.260	0.664
		250–352 μm	Normal distribution	49.7	21.82	0.194	0.856
	BA	<63 μm	Lognormal distribution	0.1	0.02	0.112	0.991
		63–150 μm	Lognormal distribution	0.09	0.04	0.276	0.644
		150–250 μm	Lognormal distribution	0.09	0.05	0.203	0.973
		250–352 μm	Lognormal distribution	0.06	0.02	0.238	0.849
Cu	*C*	<63 μm	Lognormal distribution	637.07	263.54	0.243	0.849
		63–150 μm	Lognormal distribution	438.89	184.78	0.154	0.946
		150–250 μm	Lognormal distribution	342.54	143.33	0.198	0.741
		250–352 μm	Lognormal distribution	328.75	175.01	0.504	0.063
	BA	<63 μm	Lognormal distribution	0.31	0.06	0.331	0.496
		63–150 μm	Lognormal distribution	0.31	0.13	0.161	0.824
		150–250 μm	Lognormal distribution	0.28	0.11	0.351	0.213
		250–352 μm	Lognormal distribution	0.22	0.07	0.236	0.485
Ni	*C*	<63 μm	Lognormal distribution	27.53	11.86	0.398	0.128
		63–150 μm	Lognormal distribution	23.92	7.18	0.348	0.192
		150–250 μm	Lognormal distribution	22.32	8.58	0.374	0.154
		250–352 μm	Normal distribution	21.71	8.58	0.298	0.550
	BA	<63 μm	Lognormal distribution	0.13	0.04	0.247	0.453
		63–150 μm	Lognormal distribution	0.13	0.07	0.418	0.180

		150–250 μm	Lognormal distribution	0.13	0.06	0.256	0.422
		250–352 μm	Lognormal distribution	0.11	0.06	0.691	0.051
Pb	*C*	<63 μm	Lognormal distribution	445.37	153.35	1.76	1.00
		63–150 μm	Normal distribution	425.6	231.28	0.650	0.069
		150–250 μm	Lognormal distribution	338.27	125.52	0.659	0.065
		250–352 μm	Lognormal distribution	318.83	158.29	0.631	0.096
	BA	<63 μm	Normal distribution	0.270	0.14	0.156	0.943
		63–150 μm	Normal distribution	0.21	0.09	0.220	0.784
		150–250 μm	Lognormal distribution	0.2	0.11	0.327	0.272
		250–352 μm	Normal distribution	0.13	0.04	0.401	0.321

Table S6. Probability distribution of health risk assessment parameters.

Exposure factors	Symbol (unit)	Probabilistic distribution	Parameter
Oral ingestion rate of soils	*IRs* (mg·day^{-1})	Triangular	TRI (66, 103, 161)
Exposure frequency	*EF* (day·a^{-1})	single-point input	350
Exposure duration	*ED* (a)	single-point input	6
Body weight	*BW* (kg)	Lognormal	LN (37.0, 2.98)
Average time	*AT* (days)	single-point input	(ED×365)

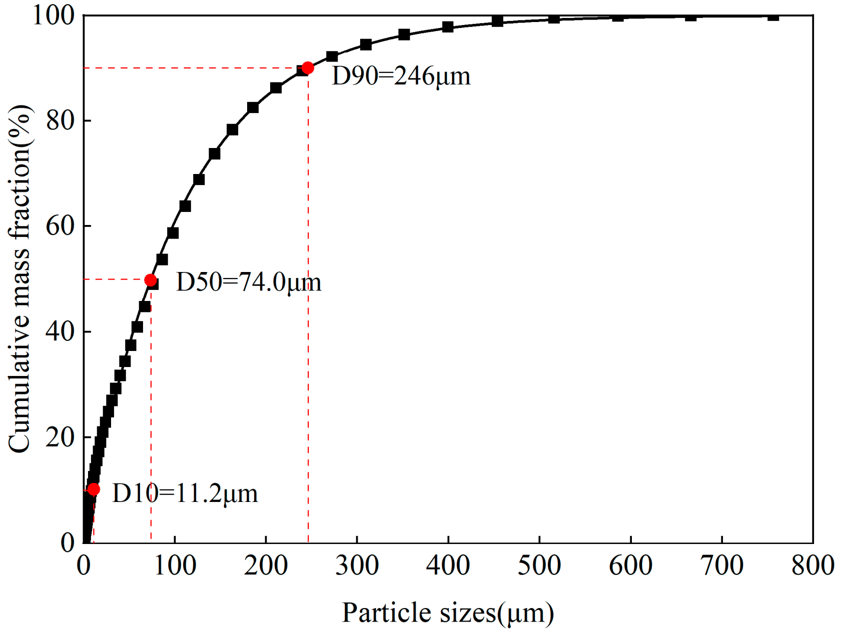

Figure S1. Cumulative mass distribution of particle sizes in soil.

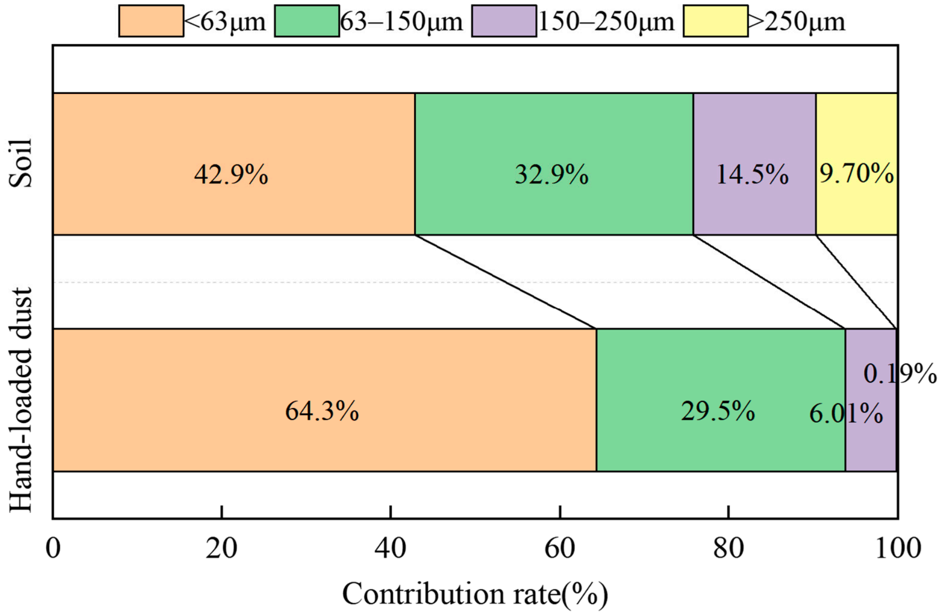

Figure S2. Stacked bar chart of particle size contribution rates for hand-loaded dust and bulk soil.

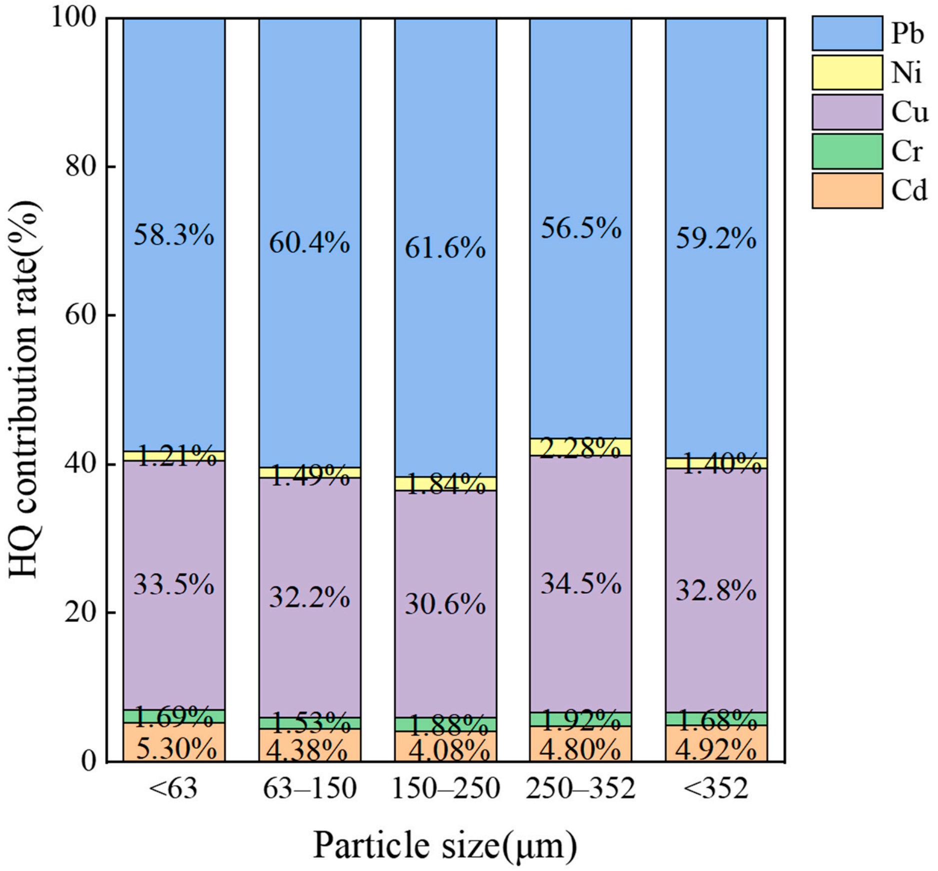

Figure S3. Contribution of soil heavy metals to total hazard quotient across different particle sizes.
